# Artificial intelligence in image-guided cervical interventional radiotherapy: a systematic review

**DOI:** 10.3389/fonc.2026.1910070

**Published:** 2026-10-15

**Authors:** Valentina Lancellotta, Angeles Rovirosa, Maria Concetta La Milia, Enrico Rosa, Bruno Fionda, Maria Vaccaro, Pierpaolo Dragonetti, Leonardo Bannoni, Rosa Autorino, Nicolò Bizzarri, Luca Russo, Benedetta Gui, Gabriella Macchia, Luca Tagliaferri, Maria Antonietta Gambacorta

**Affiliations:** 1 Fondazione Policlinico Universitario “A. Gemelli” IRCCS, Radioterapia Oncologica, Dipartimento di Diagno-stica per Immagini e Radioterapia Oncologica, Rome, Italy; 2 Radiation Oncology Department, Hospital Clínic-Universitat de Barcelona, Barcelona, Spain; 3 UOC Fisica per le Scienze della Vita, Dipartimento di Diagnostica per Immagini e Radioterapia Oncologica, Fondazione Policlinico Universitario A. Gemelli IRCCS, Rome, Italy; 4 Department of Theoretical and Applied Sciences, eCampus University, Novedrate, Italy; 5 Unità Operativa Complessa Ginecologia Oncologica, Dipartimento per la Salute Della Donna e del Bambino e Della Salute Pubblica, Policlinico Agostino Gemelli Istituto di Ricovero e Cura a Carattere Scientifico, Rome, Italy; 6 Department of Imaging and Radiation Oncology, Fondazione Policlinico Universitario A. Gemelli IRCCS, Rome, Italy; 7 Radiation Oncology Unit, Responsible Research Hospital, Campobasso, Italy; 8 Università Cattolica del Sacro Cuore Sede di Roma, Rome, Italy

**Keywords:** artificial intelligence, cervix cancer, image-guided interventional radiotherapy (modern brachytherapy), treatment personalization, workflow efficiency

## Abstract

**Background:**

Imaging-guided interventional radiotherapy (IG-IRT) is the standard of care for locally advanced cervical cancer but remains time-consuming and operator-dependent. Artificial intelligence (AI) has emerged as a promising tool to improve efficiency and reproducibility.

**Methods:**

A systematic review was conducted according to PRISMA 2020 guidelines, searching PubMed, Scopus, and Web of Science for studies published between 2010 and 2026 investigating AI-based imaging applications in cervical IRT. Eligible studies included original research evaluating segmentation (targets and organ at risk), planning, reconstruction, image registration and applicator selection.

**Results:**

Fifty-two studies were included. Deep learning–based segmentation showed variable performance across anatomical structures and studies, with Dice coefficients approaching or exceeding 0.90 for some organs at risk and target volumes in the best-performing models. Individual studies reported shorter processing times, reduced contouring variability, accurate applicator reconstruction, and potential support for applicator selection. However, the substantial methodological and clinical heterogeneity precluded direct comparisons among AI approaches.

**Conclusions:**

The available studies suggest that AI may support the automation of selected workflow steps and assist treatment-related decision-making in image-guided cervical interventional radiotherapy. While several applications have achieved high technical performance, most remain at the proof-of-concept stage, and robust external validation and prospective clinical evaluation are needed before routine clinical adoption.

## 1 Introduction

Cervical cancer represents a significant global health burden and remains among the leading causes of cancer-related morbidity and mortality in women worldwide (1, 2). In patients with locally advanced disease, the standard of care consists of external beam radiotherapy (EBRT) with concurrent chemotherapy ± immunotherapy followed by an interventional radiotherapy (IRT, modern brachytherapy) boost (3–5). IRT allows the delivery of highly conformal radiation doses directly to the tumor while sparing surrounding organs at risk (OARs), owing to the steep dose gradients inherent to intracavitary and interstitial techniques (6).

Over the past decades, cervical IRT has evolved from two-dimensional planning to image-guided IRT (IG-IRT). In this context, magnetic resonance imaging (MRI) has emerged as the gold standard for target delineation due to its superior soft tissue contrast, enabling accurate definition of tumor volumes such as the gross tumor volume (GTV) and high-risk clinical target volume (HR-CTV) (7, 8). The introduction of MRI-guided IRT has significantly improved treatment outcomes, including better local control and reduced treatment-related toxicity compared to conventional approaches (9).

Despite these advances, MRI-guided IRT remains a complex and opera-tor-dependent procedure. Critical steps such as target delineation, segmentation of OARs, applicator reconstruction, and treatment planning are time-consuming and subject to inter- and intra-observer variability (10, 11). Furthermore, anatomical changes, including organ motion and applicator displacement, may occur during treatment, potentially affecting dose distribution and treatment accuracy (12). These limitations highlight the need for advanced tools to improve reproducibility, efficiency, and standardization in clinical workflows.

In recent years, artificial intelligence (AI) has emerged as a transformative approach in oncological imaging, with applications spanning lesion detection, segmentation, and quantitative image analysis (13, 14). In radiotherapy, deep learning models—particularly convolutional neural networks (CNN)—have demonstrated high accuracy in automatic segmentation of target volumes and OARs, reducing contouring time and variability (15, 16). In the context of cervical IRT, AI-based methods are increasingly being explored to automate key steps of the workflow, including segmentation, applicator reconstruction, and treatment planning optimization (17).

Beyond the AI applications currently investigated in cervical IG-IRT, radiomics represents a promising and potentially relevant approach for extracting quantitative imaging biomarkers and supporting treatment personalization (18–20). However, no studies included in the present review directly evaluated radiomics applications in this specific clinical setting; therefore, its potential role and clinical value should be specifically investigated in future studies rather than considered a currently validated approach.

However, despite promising results, the clinical implementation of AI in cervical IRT remains limited. Current studies are often retrospective, based on small datasets, and lack external validation (17, 21). Additionally, challenges related to data heterogeneity, standardization of imaging protocols, and model generalizability must be ad-dressed before widespread clinical adoption can be achieved (22).

The aim of this systematic review is not only to summarize current applications of artificial intelligence in image-guided cervical IRT, but also to critically assess their level of clinical maturity, distinguish technical performance from demonstrated clinical benefit, and identify the evidence gaps that currently limit their translation into routine clinical practice.

## 2 Materials and methods

### 2.1 Study design and reporting standards

This systematic review was conducted in accordance with the Preferred Reporting Items for Systematic Reviews and Meta-Analyses (PRISMA 2020) guidelines (23). The methodology was defined *a priori* to ensure transparency, reproducibility, and methodological rigor. The aim of this review was to investigate the current applications of artificial intelligence in cancer imaging within the context of image-guided cervical IRT, with a focus on segmentation (targets and OARs), planning, reconstruction, image registration and applicator selection. The review protocol was not prospectively registered. Nevertheless, the eligibility criteria, study-selection process, data items, and methodological assessment procedures were defined before the formal screening and data-extraction stages.

### 2.2 Eligibility criteria

Studies were selected according to predefined inclusion and exclusion criteria. Eligible studies included patients with cervical cancer undergoing image-guided interventional radiotherapy. For the purposes of this review, AI-based methods primarily included machine-learning and deep-learning approaches. Non-learning computational approaches, such as rule-based, heuristic, clustering, or optimization methods, were considered eligible only when they were explicitly developed and presented as part of an automated intelligent decision-support, planning, or reconstruction framework relevant to the cervical IG-IRT workflow. Simple deterministic automation without an AI, adaptive, predictive, or decision-support component was not considered enough for inclusion. Eligible approaches involved supervised or unsupervised machine-learning methods, artificial neural networks, convolutional neural networks, deep-learning architectures, transformer-based models, and related data-driven computational methods. These approaches could be applied directly to CT or MRI images or to imaging-derived features used for treatment-related prediction or decision support. Studies were required to evaluate at least one of the following predefined application domains: target or organ-at-risk segmentation, image registration, treatment planning or dose prediction/optimization, applicator or catheter reconstruction, or applicator/needle selection. Studies including mixed gynecological cancer populations were eligible only when patients with cervical cancer represented an explicitly identifiable subgroup and the investigated methodology was directly applicable to cervical IG-IRT. When cervical-specific outcome data could not be extracted separately, findings from mixed cohorts were retained only as supportive technical evidence and were not interpreted as cervical-cancer-specific estimates. Only original research articles written in English were included. Review articles, editorials, letters, and conference abstracts were excluded.

### 2.3 Information sources and search strategy

A comprehensive literature search was performed in PubMed/MEDLINE, Scopus, and Web of Science. The search covered publications from January 2010 to May 2026, and the final search update was conducted in 31 May 2026. The search strategy was based on the same predefined core concepts across all three databases, namely artificial intelligence, cervical cancer, brachytherapy/interventional radiotherapy, and medical imaging. The same principal keywords and synonyms were used consistently across databases, while the search syntax and database-specific conventions were adapted according to the requirements of each platform. The core Boolean structure was as follows: (“artificial intelligence” OR “machine learning” OR “deep learning” OR “neural network”) AND (“cervical cancer” OR “cervix cancer”) AND (“brachytherapy” OR “image-guided brachytherapy” OR “interventional radiotherapy”) AND (“MRI” OR “magnetic resonance imaging” OR “CT” OR “computed tomography” OR “medical imaging”). This conceptual search structure was maintained across PubMed/MEDLINE, Scopus, and Web of Science, with minor adaptations in syntax as required by each database. No additional language filter was applied during the electronic search; English-language eligibility was assessed during the study-selection process. In addition, the reference lists of all included articles were manually screened to identify further relevant studies (**Supplementary Table S1**).

### 2.4 Study selection

The selection process was carried out in two stages. Initially, titles and abstracts were independently screened by two reviewers to identify potentially eligible studies. Subsequently, the full texts of the selected articles were assessed for eligibility based on the predefined criteria. Any disagreements between reviewers were resolved through discussion, and when necessary, consultation with a third reviewer was undertaken to reach consensus. The overall study selection process was documented using a PRISMA flow diagram.

### 2.5 Data collection and data items

Data extraction was performed independently by two reviewers using a standardized data extraction form to ensure consistency. For each included study, detailed information was collected regarding study characteristics, including authorship, year of publication and study design. Information on imaging modalities and AI methodologies was also extracted, including the type of imaging used (MRI, CT) and the specific AI approach applied. Performance metrics reported in the studies were recorded.

### 2.6 Risk of bias assessment

The methodological quality and risk of bias of the included studies were independently evaluated by two reviewers using prespecified task-specific frameworks. Studies of automated segmentation, applicator or catheter reconstruction, and image registration were appraised using the four-domain structure of QUADAS-2, supplemented by the AI-specific methodological considerations proposed by Sounderajah et al. as part of the QUADAS-AI initiative (24). The four original QUADAS-2 domains—patient selection, index test, reference standard, and flow and timing—were retained. The signalling questions were operationalized for AI-based imaging tasks in accordance with the AI-specific methodological considerations proposed within the QUADAS-AI framework (24). The patient-selection domain addressed the source, size, and quality of the input data, patient eligibility criteria, use of open-source datasets, reporting of training, validation, and test-set allocation, image preprocessing, and scanner information. The index-test domain assessed whether external validation was performed. The reference-standard domain assessed whether the reference standard was likely to correctly classify the target condition. The flow-and-timing domain assessed whether the time interval between the index test and the reference standard was appropriate.

Although segmentation, reconstruction, and image registration are not conventional diagnostic-accuracy tasks, this framework was selected because these studies compare AI-generated imaging outputs with clinician-defined or clinically approved reference standards and share methodological concerns related to input data, evaluation of the AI method, the appropriateness of the reference standard, and the relationship between the index test and reference standard. The resulting judgments were therefore interpreted as task-specific methodological assessments rather than formal diagnostic-accuracy assessments.

Studies of treatment planning, dose prediction or optimization, and applicator selection were evaluated using the published 2025 version of PROBAST+AI (25). The four domains assessed were participants and data sources, predictors, outcome, and analysis. The corresponding PROBAST+AI signalling questions were applied to assess risk of bias and concerns regarding applicability. Domain-level judgments were assigned in accordance with the PROBAST+AI guidance. Assessments were performed independently by two reviewers, and disagreements were resolved through discussion or, when necessary, consultation with a third senior reviewer.

### 2.7 Effect measures

The primary effect measures considered in this review were extracted according to the specific AI task under investigation. Segmentation studies were primarily evaluated using geometric metrics, including the Dice Similarity Coefficient (DSC), Hausdorff Distance/95th percentile Hausdorff Distance (HD/HD95), Average Surface Distance/Average Symmetric Surface Distance (ASD/ASSD), Mean Surface Distance (MSD), Jaccard Index (JI), Intersection over Union (IoU), Surface Dice, and other overlap-based measures. Registration studies were assessed using geometric agreement and deformation metrics, including Dice coefficient, Jaccard Index, Average Symmetric Surface Distance, Mean Distance Error, and Hausdorff Distance. Reconstruction studies were evaluated using geometric accuracy metrics, such as Dice coefficient, Hausdorff Distance, tip and shaft localization errors, reconstruction variability, and dwell position deviations. Planning studies were assessed using dosimetric and predictive metrics, including dose received by 90% of the target volume (D90), dose received by 98% of the target volume (D98), volume receiving 100% of the prescribed dose (V100), volume receiving 150% of the prescribed dose (V150), volume receiving 200% of the prescribed dose (V200), dose to the most exposed 2 cm³ (D2cc), dose to the most exposed 1 cm³ (D1cc), maximum dose (Dmax), Equivalent Dose in 2 Gy Fractions (EQD2), mean absolute error (MAE), mean squared error (MSE), gamma pass rate, and correlation coefficients. Applicator selection studies were evaluated using classification metrics, including accuracy, sensitivity, specificity, precision, F1-score, and area under the receiver operating characteristic curve (AUC), as well as dosimetric indicators of target coverage and organ-at-risk sparing.

Reported values were extracted directly from the included studies and synthesized qualitatively according to the corresponding AI task. A qualitative synthesis of the results was performed. The included studies were analyzed descriptively, focusing on the clinical application of AI and its reported performance. A formal meta-analysis was not conducted. Instead, findings were summarized to identify common trends, strengths, and limitations in the current literature.

### 2.8 Assessment of reporting bias

Reporting bias and selective outcome reporting were assessed by comparing the outcomes specified in the methods and results sections of each included study and by evaluating the completeness of reporting of clinically relevant outcomes. Particular attention was paid to whether studies reported both technical performance metrics and clinically meaningful endpoints, such as dosimetric parameters, treatment planning outcomes, or physician acceptability assessments.

### 2.9 Certainty of evidence

Because the included studies evaluated heterogeneous technical tasks and outcomes, certainty was assessed separately for each application domain—segmentation, treatment planning, reconstruction, applicator selection, and image registration—rather than by combining all studies into a single certainty rating. Within each application domain, studies reporting conceptually related endpoints were considered as one body of evidence. These endpoints included geometric agreement for segmentation, reconstruction, and registration; dosimetric accuracy and plan quality for treatment-planning studies; and classification performance and dosimetric consequences for applicator-selection studies.

A GRADE-informed approach was used to assess five domains: risk of bias, inconsistency, indirectness, imprecision, and publication bias. Risk of bias was judged using the corresponding methodological assessment: the QUADAS-2 assessment supplemented by the AI-specific methodological considerations proposed within the QUADAS-AI framework for segmentation, reconstruction, and image-registration studies, and PROBAST+AI 2025 for planning and applicator-selection studies. Certainty was downgraded for risk of bias when important methodological limitations affected a substantial proportion of the contributing studies, particularly inappropriate patient selection, non-independent reference standards, data leakage, inadequate data partitioning, overfitting, or lack of independent evaluation.

Inconsistency was judged according to the direction and magnitude of between-study variability in reported performance, considering differences in imaging modality, anatomical structure, AI architecture, reference standard, performance metric, and institutional setting. Because quantitative pooling was inappropriate, inconsistency was assessed qualitatively rather than using a I² statistic. Indirectness was considered present when the available evidence relied predominantly on surrogate technical metrics rather than clinical endpoints, included mixed gynecological populations without separately extractable cervical-cancer results, or was derived from narrowly selected single-center or single-vendor datasets that did not fully represent the intended clinical setting. Imprecision was judged from the number and size of the contributing studies, the amount of evaluation data, the width or availability of confidence intervals, and the stability of the reported performance estimates. Formal funnel-plot assessment was not performed because of the small number of studies within several application domains and the marked heterogeneity of outcomes and metrics. Publication bias was assessed qualitatively by considering the predominance of small, retrospective, single-center proof-of-concept studies, the availability of independent replications, and the possible underrepresentation of negative or inconclusive findings. Formal funnel-plot assessment was not performed because of the marked heterogeneity of the included endpoints and the limited number of studies within several application domains.

Final certainty was categorized as high, moderate, low, or very low. No upgrading criteria were applied. The reasons for each final certainty rating were recorded by application domain in the corresponding Summary of Findings tables.

## 3 Results

### 3.1 Study selection

The database search identified 353 records. After the removal of 127 duplicates, 226 records remained for title and abstract screening. Of these, 158 records were excluded because they did not meet the eligibility criteria or were not relevant to the review objective. The full texts of the remaining 68 articles were assessed for eligibility. Following full-text evaluation, 16 studies were excluded for specific reasons related to the predefined eligibility criteria, as detailed in the PRISMA flow diagram and **Supplementary Table S2**. Ultimately, 52 studies were included in the qualitative synthesis (26–77) (**Figure 1**).

**Figure 1:**
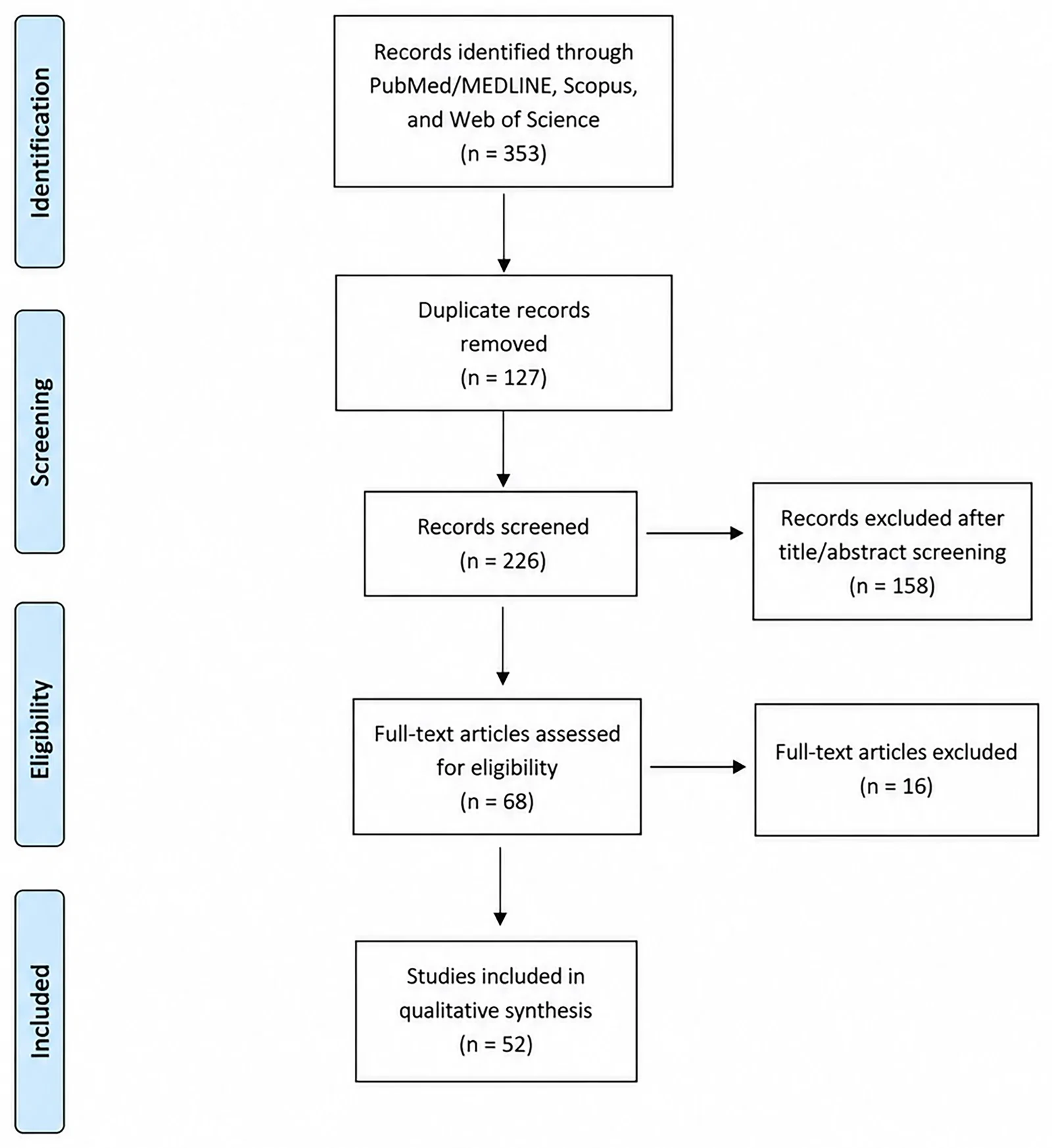
PRISMA flow-chart.

### 3.2 Study characteristics and results of individual studies

#### 3.2.1 AI-based segmentation in high dose rate (HDR)-IRT for cervical cancer.

More than 2,951 patients were included across the selected studies (**Table 1**).

**Table 1:** Literature summary of AI-based segmentation in HDR-IRT for cervical cancer.

Study	Design/Imaging	AI method	Target	Key findings
Cao et al. (2025) (26)	Retrospective; CT	MDA-TransUNet	HR-CTV, OARs	DSC up to 94.5% for OARs and 82.4% for HR-CTV; no significant dosimetric differences vs manual contours; ~26-fold faster contouring.
Cao et al. (2022) (27)	Retrospective; CT/MRI	Asymmetric Dual-Path 3D CNN	HR-CTV	Best model achieved DSC 0.76 (0.61–0.85), sensitivity 0.81, precision 0.83, 95-HD 5.99 mm, and AUC 0.86; performance was comparable to expert intra-operator agreement and superior to inter-operator variability.
Huang et al. (2023) (28)	Retrospective; CT	Mnet_Im	CTV	Best overall segmentation performance (ASD 0.69; HD95 3.20 mm), outperforming U-Net, UNet++, DeepLabV3+, M-Net, and other baseline models.
Kano et al. (2021) (29)	Retrospective; MRI	2D U-Net + 3D U-Net	GTV	Median DSC 0.83 (mean 0.77; range 0.13–0.93) and median HD 4.7 mm; accuracy decreased in tumors <40 mm, tumors with necrosis, and retained uterine hematoma.
Kim et al. (2024) (30)	Retrospective; MRI	DCT-UNet	HR-CTV, OARs	DSC 0.932 ± 0.032 for bladder, 0.786 ± 0.090 for rectum, 0.663 ± 0.180 for sigmoid, and 0.741 ± 0.076 for HR-CTV; performance comparable to human observers; segmentation time 12.59 ± 0.79 s.
Lei et al. (2025) (31)	Retrospective; CT	Intuitive U-Net + DUCK-Transformer	HR-CTV, OARs	DSC 0.79 ± 0.06 for HR-CTV and 0.63–0.83 for OARs; mean HR-CTV D90 difference 0.42 ± 1.17 Gy, with no significant dosimetric differences vs manual contours.
Mohammadi et al. (2021) (32)	Retrospective; CT	ResU-Net CNN	OARs	DSC 93.0–96.6%, outperforming U-Net, with minimal dosimetric differences; automatic contouring completed in ~1.5 s/patient.
Ni et al. (2024) (33)	Retrospective; MRI	nnU-Net	OARs	Transfer learning improved model generalizability and restored accurate OAR segmentation across unseen scanners and applicators.
Oliva et al. (2025) (34)	Retrospective; MRI	nnU-Net	OARs	Clinically acceptable OAR segmentation with high dosimetric agreement between manual and predicted contours and consistent performance across applicator types, OAR volumes, and contourers.
Peng et al. (2026) (35)	Retrospective; CT	SPSeg	HR-CTV	SPSeg-Dual outperformed nnU-Net and SAM-Med3D across all multicenter test sets, with DSC up to 0.95 and HD95 as low as 2.1 mm; contouring time reduced by >84% and inter-observer agreement improved.
Rigaud et al. (2021) (36)	Retrospective; CT	DeepLabV3+ (2D) + 3D U-Net	CTV, OARs	Robust internal and external validation (CTV DSC 0.81–0.88), with >90% target coverage using auto-segmented contours, enabling online adaptive optimization.
Rodríguez Outeiral et al. (2023) (37)	Retrospective; MRI	3D nnU-Net	GTV	Median DSC ~0.70–0.75; ΔD90 and ΔD98 were significantly lower for smaller tumors (0.3–2.8 cc) than for the largest tumors (12.1–52.9 cc) (p<0.01).
Simões et al. (2024) (38)	Retrospective; MRI	CNN + fine-tuning	CTV	Patient-specific models improved performance and reduced contouring time; fine-tuning improved DSC and dosimetric differences were clinically negligible.
Sun et al. (2024) (39)	Retrospective; CT/MRI	AGSC-GAN + GSN	CTV	Multimodal CT/AI-generated MRI framework achieved DSC 0.95 and IoU 0.90, outperforming FCN, U-Net, Attention U-Net, DeepLabV3+, and PSPNet.
Takahashi et al. (2026) (40)	Retrospective; CT	nnU-Net	OARs	High agreement with manual contours; automatic contouring required ~30 s/case and maintained performance in the presence of interstitial needles.
Tan et al. (2025) (41)	Retrospective; CT	ARGNet	CTV	Outperformed U-Net, GLIA-Net, UNETR, and nnU-Net; incorporation of interstitial needle information significantly improved accuracy and robustness (p<0.05).
Wang et al. (2025) (42)	Retrospective; CT	LLM (ChatGPT)	HR-CTV, OARs	LLM-guided prompting improved HR-CTV and OAR segmentation accuracy compared with baseline models, with rapid inference (<0.5 s/scan).
Xue et al. (2024) (43)	Retrospective; CT/MRI	3D Prompt-ResUNet	HR-CTV, OARs	Outperformed nnUNet and SAM-Med3D, achieving expert-level contouring performance, reducing interobserver variability, and shortening treatment times.
Yoganathan et al. (2022) (44)	Retrospective; MRI	ResNet50	CTV, OARs	Mean DSC ~0.72 overall, with higher performance for OARs than target volumes; InRN performed slightly better than ResNet50.
Zabihollahy et al. (2022) (45)	Retrospective; MRI	3D U-Net + Attention U-Net + 3D Block-based U-Net	CTV	Fully automated MRI-based CTV segmentation achieved DSC 0.85 ± 0.03 and HD95 3.70 ± 0.35 mm, outperforming alternative architectures and providing uncertainty maps for contour review.
Zaffino et al. (2019) (46)	Retrospective; MRI	3D U-Net CNN	Catheter	Accurate catheter detection with mean distance error 2.0 mm, DSC 0.60, and false-negative rate 1.5%.
Zang et al. (2025) (47)	Retrospective; CT	AM-UNet	HR-CTV, OARs	DSC 0.73–0.94; 93.1% of HR-CTV contours were clinically acceptable and dosimetric differences were generally <1% vs manual contours.
Zhang et al. (2020) (48)	Retrospective; CT	DSD-UNET (3D CNN)	HR-CTV, OARs	Simultaneous HR-CTV/OAR segmentation achieved DSC up to 86.9%.
Zhao et al. (2025) (49)	Retrospective; CT	C2FAU-Net	OARs	Mean DSC 89.7%, HD95 3.61 mm, and ASSD 1.02 mm; delineation time reduced from 17.85 to 7.54 min, with no significant dosimetric differences vs manual contours.
Zhu et al. (2024) (50)	Retrospective; CT	SERes-U-Net	HR-CTV, OARs	Mean DSC 80.8% for HR-CTV, 91.9% for bladder, 85.2% for rectum, 60.4% for sigmoid, and 82.8% for bowel loops; most contours were clinically acceptable with no significant dosimetric differences vs manual contours.

2D, Two dimensional; AI, artificial intelligence; ASD, Average Surface Distance; ASSD, average symmetric surface distance; AUC, area under curve; CNN, convolutional neural networks; CT, computed tomography; CTV, Clinical target volume; DSC, Dice similarity coefficient; DTA, Distance-to-Agreement; Gy, gray; GTV, Gross tumor volume; HD, Hausdorff distance; HD95, hausdorff distance 95th percentile; HDR, high dose rate; HR-CTV, high risk clinical target volume; IoU, Intersection over Union; LLMs, large language models; MRI, magnetic resonance imaging; OARs, organs at risk; s, second (s); ΔD90, difference in D90; ΔD98, difference in D98.

##### 3.2.1.1 CTV segmentation

Automatic target-volume segmentation was investigated using CNN-, U-Net-, and transformer-based architectures for HR-CTV, CTV, and GTV delineation (27–29, 35, 37–39, 41, 45). Performance ranged from Dice coefficients of approximately 0.65–0.95. Among the reported results, the multimodal GSN framework achieved a Dice coefficient of 0.947 (39), while SPSeg-Dual achieved Dice coefficients ranging from 0.913 to 0.948 and showed statistically higher performance than nnU-Net and SAM-Med3D within that study (p < 0.001) (35). Significant improvements over comparator models were also reported for the dual-path CT/MRI CNN (p < 0.01) (27), ARGNet (p < 0.05) (41), and the cascaded MRI framework (p < 0.05) (45). Additional studies demonstrated promising performance using Mnet_IM (28), Diffusion-Weighted Imaging (DWI)-based U-Net ensembles (29), MRI-based nnU-Net (37), patient-specific adaptive models (38), and multimodal CT/MRI approaches (39).

##### 3.2.1.2 OAR segmentation

OAR auto-segmentation was evaluated using ResU-Net, nnU-Net, transfer-learning strategies, and attention-based U-Net architectures (32–34, 40, 49). Bladder segmentation generally showed higher reported Dice coefficients than the other evaluated organs, ranging from 0.92 to 0.96 across individual studies. Transfer learning significantly improved performance across bladder, rectum, sigmoid, and bowel structures after fine-tuning (p = 0.012–<0.001) (33). ResU-Net demonstrated significantly improved sigmoid dosimetry compared with standard U-Net (p < 0.05) (32). High geometric and dosimetric agreement with manual contours was also reported for nnU-Net–based approaches (34, 40). C2FAU-Net achieved a mean DSC of 89.7% while significantly reducing contouring time without clinically relevant dosimetric differences compared with manual contours (49).

##### 3.2.1.3 Combined CTV and OAR segmentation

Several studies simultaneously segmented target volumes and OARs using MDA-TransUNet, DCT-UNet, dual-stage CNN, DeepLabV3+/3D U-Net frameworks, large language model (LLM)-guided models, Prompt-ResUNet, AM-UNet, DSD-UNET, and SERes-U-Net architectures (26, 30, 31, 36, 42–44, 47, 48, 50). MDA-TransUNet achieved Dice values up to 94.5% for OARs and 82.4% for HR-CTV, with no significant dosimetric differences versus manual contours (26). DCT-UNet demonstrated performance comparable to human observers while substantially reducing contouring time (30). Significant improvements were reported for dual-stage CNN (p < 0.005) (31), LLM-guided segmentation (overall DSC p = 0.014, HD95 p < 0.001) (42), AM-UNet (p < 0.01–0.02) (47), SERes-U-Net (p = 0.012–0.047) (50), and 2.5D models for HR-CTV and intermediate risk clinical target volume (IR-CTV) segmentation (p = 0.031–0.001) (44). DeepLabV3+ and three-dimensional (3D) U-Net models reported favorable performance in internal and external validation cohorts, enabling online adaptive optimization with high target coverage (36). Prompt-ResUNet achieved Dice values of 0.92 for HR-CTV, 0.93 for bladder, and 0.87 for rectum, outperforming nnUNet and SAM-Med3D (43).

##### 3.2.1.4 Catheter segmentation

Catheter segmentation was addressed by Zaffino et al. using a 3D CNN on intraoperative MRI, achieving a mean Dice coefficient of 0.60 ± 0.17, a mean distance error of 2.0 ± 3.4 mm, and a false-negative detection rate of 1.5% (46).

Among the segmentation approaches evaluated, individual studies reported promising performance for SPSeg-Dual in CTV/HR-CTV delineation (35), transfer-learning approaches in OAR segmentation (33), and AM-UNet, Prompt-ResUNet, and MDA-TransUNet in combined target and OAR segmentation (26, 43, 47). Within-study comparisons reported statistically significant improvements over selected benchmark architectures for SPSeg-Dual, transfer-learning frameworks, and AM-UNet (33, 35, 47). However, differences in datasets, anatomical targets, imaging modalities, comparators, and validation strategies preclude direct ranking of these architectures.

#### 3.2.2 AI-based planning in HDR-IRT for cervical cancer

AI applications in cervical cancer interventional radiotherapy planning have focused on dose prediction, automated optimization, plan quality assurance, and adaptive planning. Multi-objective optimization with BRachytherapy via artificially Intelligent GOMEA-Heuristic based Treatment Planning (BRIGHT) improved protocol compliance, achieving 100% adherence to EMBRACE-II limits versus 88.9% in clinical plans and significantly better OAR sparing (p = 1.08 × 10^-7^) while maintaining comparable target coverage (p = 0.12) (51). Deep-learning frameworks were developed for plan approval prediction (52), voxel-wise dose prediction (53, 57, 59), dwell-time optimization (56, 58, 63), and independent plan verification (55), generally demonstrating high agreement with clinical plans. Dose prediction accuracy was particularly high for SE_AN, with mean absolute errors of 0.37 Gy for HR-CTV D90 and 0.23–0.28 Gy for OAR D2cc values (57), while attention-gated 3D U-Net models generated complete dose distributions within 5 seconds (53). Knowledge-based planning approaches reduced OAR doses by up to 10.7% for rectum D2cc and 10.3% for bladder D2cc (54), whereas automated planning systems generated clinically acceptable plans in 1.4–3.5 minutes with no significant differences in HR-CTV or OAR dosimetry compared with manual planning (58, 63). Fully automated adaptive planning further improved treatment quality, with automated plans preferred in 60/63 cases and achieving significantly lower bladder (p = 0.05) and rectum (p = 0.04) doses while increasing the proportion of patients reaching CTVHR D90 ≥ 90 Gy (14 vs 9 patients) (60). Similarly, the rule-based BiCycle system significantly reduced bladder, rectum, sigmoid, and bowel doses while being preferred by physicians in 75–76% of cases (62). Machine-learning quality-assurance tools based on overlap volume histogram (OVH) analysis (61), neural-network OAR dose prediction (64), and automated plan verification (55) also reported promising predictive performance in individual studies.

Among the planning approaches evaluated, individual studies reported promising results for BRIGHT (51), automated adaptive planning systems (60, 62), and deep-learning-based dose-prediction and optimization frameworks (56–59, 63). Some studies reported clinically acceptable plans, improved OAR sparing, or shorter planning times compared with their respective conventional approaches. However, the heterogeneity of datasets, endpoints, comparators, and validation methods precludes direct comparisons or ranking of these approaches. Further prospective and independent external validation is required (**Table 2**).

**Table 2:** Literature summary of AI-based planning in HDR-IRT for cervical cancer.

Study	Design/Imaging	AI method	Target	Key findings
Dickhoff et al. (2024) (51)	Retrospective; CT/MRI	BRIGHT, MO-RV-GOMEA	HR-CTV, IR-CTV, GTVres, OARs	Generated high-quality IRT plans, achieving EMBRACE-II constraints more frequently than clinical plans (100% vs 88.9% limits; 83.6% vs 47.2% aims), while enabling rapid re-optimization (~30 s).
Gao et al. (2024) (52)	Retrospective; CT	Adversarial Deep Learning (Dose Prediction Network + Plan-Approval Probability Network)	HR-CTV, OARs	Predicted physician plan approval probability. For 20% dose perturbations, PPN achieved accuracy 0.70, sensitivity 0.74, specificity 0.65, and AUC 0.74. DPN predicted dosimetric metrics with relative errors of 11.5% (bladder), 8.2% (rectum), 7.1% (sigmoid), and 10.2% (CTV).
Gautam et al. (2024) (53)	Retrospective; CT	Attention-Gated 3D U-Net	HR-CTV, OARs	HR-CTV D90 prediction error: 0.46 ± 2.44 Gy (8.14%); bladder D2cc error: 0.22 ± 1.22 Gy (3.62%); rectum D2cc error: 0.21 ± 1.06 Gy (2.20%); sigmoid D2cc error: −0.03 ± 0.54 Gy (1.13%). Full 3D dose prediction completed in <5 s/patient.
Gautam et al. (2025) (54)	Retrospective; CT	Knowledge-Based Planning (OVH-based linear prediction model)	HR-CTV, OARs	Improved plan optimization by accurately predicting OAR doses, reducing bladder, rectum, and sigmoid D2cc by up to 10.3%, 10.7%, and 6.9%, respectively.
Fan et al. (2021) (55)	Retrospective; CT	Inception-based CNN + Encoder Network	Dwell positions and dwell times	Predicted dwell-position coordinates differed by approximately 1 pixel (~0.5 mm) from TPS values; predicted dwell times showed <2% relative deviation, demonstrating accurate independent verification of HDR-IRT plans.
Kallis et al. (2023) (56)	Retrospective; CT	3D U-Net + COBYLA	HR-CTV, OARs	Generated clinically acceptable treatment plans with high agreement with clinical plans (DSC 0.91), enabling automated dose prediction and plan optimization in ~1.6 min/case.
Li et al. (2023) (57)	Retrospective; CT	SE_AN (Squeeze-and-Excitation Attention Network based on Cascaded U-Net)	HR-CTV, OARs	The model achieved MAEs of 0.37 ± 0.25 for HR-CTV D90, 0.23 ± 0.14 for bladder D2cc, and 0.28 ± 0.20 for rectum D2cc, demonstrating performance comparable to clinically approved plans.
Mitra et al. (2026) (58)	Retrospective; CT	COBYLA	HR-CTV, OARs	AI-generated plans achieved dosimetric quality comparable to clinical plans, were produced in 1.4 min, and were rated clinically acceptable, with ~50% preferred or considered equivalent by experts.
Moore et al. (2024) (59)	Retrospective; CT	3D Cascade U-Net	3D dose distribution	Fine-tuned models achieved MAE of 2.69–5.36%, gamma pass rates of 83–91%, and DSC of 0.88–0.92, demonstrating accurate applicator-specific 3D dose prediction and feasibility of automated IRT planning.
Oud et al. (2020) (60)	Retrospective; MRI	Erasmus-iCycle	HR-CTV, OARs	Automated planning improved achievement of HR-CTV D90 >90 Gy (14/16 vs 9/16 clinical plans), reduced bladder and rectum D2cc, and generated plans in 20.5 s, with 62/63 plans rated equal or superior to clinical plans.
Reijtenbagh et al. (2022) (61)	Retrospective; MRI	Random Forest + OVH	OARs	Accurately predicted OAR D2cm³ values with robust cross-institutional generalizability (MSE 0.13–0.40 Gy), enabling AI-based quality assurance in cervical IRT planning.
Rossi et al. (2025) (62)	Retrospective; MRI	BiCycle	CTV + OARs	AI-generated plans achieved target coverage comparable to manual plans while significantly reducing OAR doses, with optimization completed in 1.6 min. Physicians preferred AI plans in 75–76% of externally validated cases.
Truong et al. (2026) (63)	Retrospective; CT	3D Cascade U-Net	HR-CTV, OARs	Generated clinically comparable plans (dose MAE 3.8%; dwell-time error 10.3 s) through a fully automated planning workflow, with plan generation completed in 3.5 min.
Zhang et al. (2023) (64)	Retrospective; CT	ANN	OARs	The ANN predicted OAR doses with R values of 0.801, 0.921, and 0.929 for bladder, rectum, and sigmoid colon, respectively, and mean ΔD2cc/D90 values of 0.052, 0.040, and 0.041.

AI, artificial intelligence; ANN, artificial neural network; AUC, area under the curve; CNN, convolutional neural network; CT, computed tomography; CTV, clinical target volume; D2cc, dose to the most exposed 2 cm³; D90, dose received by 90% of the target volume; ΔD2cc/D90, difference in D2cc/D90; DPN, dose prediction network; DSC, Dice similarity coefficient; GTVres, residual gross tumor volume; Gy, gray; HDR, high dose rate**;** HR-CTV, high-risk clinical target volume; IR-CTV, intermediate-risk clinical target volume; IRT, interventional radiotherapy; MAE, mean absolute error; MRI, magnetic resonance imaging; MSE, mean squared error; OARs, organs at risk; OVH, overlap volume histogram; PPN, plan-approval probability network; s, second(s); TPS, treatment planning system.

#### 3.2.3 AI-based reconstruction in HDR-IRT for cervical cancer

AI-based image reconstruction studies, including more than 160 patients, have primarily focused on automated applicator, catheter, and needle digitization in CT- and MRI-guided cervical interventional radiotherapy. Several automated reconstruction studies reported submillimeter geometric accuracy and limited dosimetric differences compared with manual digitization. Thresholding and clustering algorithms achieved contour discrepancies of only 0.3 mm and dosimetric differences ≤ 1% in HDR tandem-and-ovoid applications (65), while deep learning–based approaches reported DSC values of 0.88–0.93, Hausdorff distances generally below 1 mm, and negligible differences in HR-CTV and OAR dose metrics (48, 66, 67, 69, 70). In a mixed gynecological cancer cohort that included patients with cervical cancer, MRI-based catheter reconstruction reduced reconstruction time from 46 ± 10 to 11 ± 1 minutes (68). Among the reconstruction approaches evaluated, individual studies reported promising performance for attention-gated CNN (69), nnU-Net-based models (70), and DSD-UNET approaches (48). These studies reported accurate automatic digitization and limited dosimetric differences compared with manual reconstruction. However, the absence of independent external validation and the heterogeneity of the evaluated tasks preclude conclusions regarding the relative superiority of these approaches (**Table 3**).

**Table 3:** Literature summary of AI-based reconstruction in HDR-IRT for cervical cancer.

Study	Design/Imaging	AI method	Target	Key findings
Deufel et al. (2020) (65)	Retrospective; CT	Image thresholding + HDBSCAN	Applicator	Automated applicator digitization showed excellent agreement with manual reconstruction (mean contour disagreement 0.3 mm, HD ≤1.0 mm, submillimeter tip agreement), with ≤1% differences in DVH parameters and execution time <30 s.
Hu et al. (2021) (66)	Retrospective; CT	U-Net	Applicator	Achieved accurate and efficient applicator reconstruction (DSC 0.89, HD95 1.66 mm) with minimal dosimetric impact versus manual reconstruction (HR-CTV D90 difference 0.29%; OAR D2cc ≤2.64%) and reconstruction time of 17 s.
Jung et al. (2020) (67)	Retrospective; CT	U-Net	Needles	In a mixed gynecological cancer cohort including cervical cancer patients, automatic needle digitization achieved DSC 0.93, mean HD ≈0.71 mm, mean tip position difference ≈0.63 mm, and completed digitization in approximately 5 min per case, demonstrating clinically acceptable accuracy and efficiency.
Shaaer et al. (2022) (68)	Retrospective; MRI	2D U-Net	Catheter	Deep-learning-assisted reconstruction achieved high catheter accuracy (mean error <1 mm), with >98% of dwell positions within 2 mm, and reduced reconstruction time from 46 to 11 minutes.
Wang et al. (2024) (69)	Retrospective; CT	Attention-Gated Deep Learning	Needles	The attention-gated model improved needle localization and digitization accuracy compared with standard 3D U-Net, achieving clinically acceptable dosimetric agreement while reducing planning time by ~93% (1–2 s per patient).
Xie et al. (2023) (70)	Retrospective; CT	CNN based on nnU-Net	Needles	The DL-based method achieved accurate interstitial needle localization with no significant dosimetric differences versus manual reconstruction, supporting automated CT-guided applicator reconstruction.
Zhang et al. (2020) (48)	Retrospective; CT	DSD-UNET (3D CNN)	HR-CTV, OARs	DSD-UNET enabled highly accurate applicator reconstruction, achieving submillimeter channel localization errors (HD <1 mm).

2D, Two dimensional; CNN, convolutional neural network; CT, computed tomography; D2cc, dose to the most exposed 2 cm³; D90, dose received by 90% of the target volume; DL, deep learning; DSC, Dice similarity coefficient; DVH, dose-volume histogram; HD, Hausdorff distance; HD95, 95th percentile Hausdorff distance; HDR, high dose rate; HR-CTV, high-risk clinical target volume; IRT, interventional radiotherapy; mm, millimeter; MRI, magnetic resonance imaging; OARs, organs at risk; s, second(s).

#### 3.2.4 AI-based applicator selection in HDR-IRT for cervical cancer

AI-based applicator selection studies included more than 260 patients and reported preliminary findings regarding the potential of machine learning to support applicator- and needle-selection decisions (71–74). In one study, a deep-learning approach achieved accuracies of 92.1–96.0% for selecting between Syed and tandem-and-ovoid applicators, 90.9% sensitivity, and 97.4% specificity (71). Machine-learning models for selecting intracavitary versus hybrid intracavitary/interstitial (IC/IS) techniques achieved accuracies up to 0.849 and AUCs of 0.903, outperforming conventional volumetric criteria, although without statistically significant differences (72). AI-assisted 3D-printed templates improved HR-CTV coverage, reduced bladder and rectum doses, and enhanced plan quality compared with free-hand insertion (73), while prospective validation demonstrated that machine learning (ML)-guided needle selection achieved 82.5% accuracy and reduced OAR doses by an average of 2.0 Gy (74). Among the approaches evaluated, individual studies reported promising performance for deep-learning-based applicator classification (70) and machine-learning-guided applicator or needle selection (72, 74). These findings suggest potential for clinical decision support, but the small number of studies, heterogeneous decision tasks, and limited independent validation preclude comparisons among approaches and require further prospective and multicenter evaluation (**Table 4**).

**Table 4:** Literature summary of AI-based applicator selection in HDR-IRT for cervical cancer.

Study	Design/Imaging	AI method	Target	Key findings
Jiang et al. (2025) (71)	Retrospective; CT	3D CNN	CTV, OARs, central tandem	Final DL model achieved 96.0% accuracy, 90.9% sensitivity, and 97.4% specificity for selecting Syed vs T&O applicators.
Kajikawa et al. (2025) (72)	Retrospective; CT/MRI	SVC, KNN, Naïve Bayes, Random Forest, Voting Ensembles + mRMR feature selection	HR-CTV, OARs, tandem applicator geometry	Logistic Regression was the best-performing model (accuracy 0.849 ± 0.023; AUC 0.903 ± 0.033). Maximum HR-CTV–tandem distance and minimum bladder short-axis length were the most important predictors for selecting ICIRT versus IC/ISIRT.
Li et al. (2026) (73)	Retrospective; CT	CNN	HR-CTV, OARs	AI-guided 3D-printed templates improved HR-CTV coverage (V100 90.19% vs 88.89%, p=0.011; D98 5.67 vs 5.55 Gy, p=0.044), reduced bladder D2cc (4.92 vs 5.10 Gy, p=0.004) and rectum D2cc (3.95 vs 4.24 Gy, p=0.010), improved CI, HI and OI, and required no intraoperative needle adjustment.
Stenhouse et al. (2024) (74)	Prospective; MRI	Machine Learning model	Applicator	Accurately supported applicator and needle selection (70% and 82.5%, respectively), improving identification of IC/IS candidates and reducing OAR doses by ~2 Gy.

3D, three dimensional; AI, artificial intelligence; AUC, area under the curve; CI, confidence index; CNN, convolutional neural network; CT, computed tomography; CTV, clinical target volume; D2cc, dose to the most exposed 2 cm³; D98, dose received by 98% of the target volume; DL, deep learning; Gy, gray; HI, Homogeneity Index; HDR, high dose rate; HR-CTV, high-risk clinical target volume; ICIRT, intracavitary interventional radiotherapy; IC/ISIRT, hybrid intracavitary/interstitial interventional radiotherapy; KNN, k-nearest neighbors; MRI, magnetic resonance imaging; mRMR, minimum redundancy maximum relevance; OARs, organs at risk; OI, Overdose Index; SVC, support vector classifier; T&O, tandem and ovoids; V100, volume receiving 100% of the prescribed dose.

#### 3.2.5 AI-based image registration in HDR-IRT for cervical cancer

AI-based image registration studies included more than 200 patients and focused on applicator registration, deformable image registration, and prediction of applicator-induced anatomical deformation (75–77). A neural network–assisted registration framework achieved applicator dwell-position errors as low as 0.7 ± 0.5 mm and completed MRI-based registration in less than 30 seconds (75). Deep learning–based deformable image registration (DIR) further demonstrated high registration accuracy, with Dice coefficients of 0.89 for the cervix, 0.96 for the bladder, and 0.93 for the rectum, outperforming the conventional SimpleElastix algorithm across all evaluated metrics (76). In addition, one study reported that an AI-based model predicted applicator-induced uterine deformation from pre-interventional radiotherapy MRI, achieving Dice coefficients of 94.1% for uterine segmentation and 88.1% for deformation prediction, with a mean uterine displacement of 25.0 mm (77). Individual studies reported promising results for deep-learning-based deformable registration and deformation prediction (76, 77). These approaches showed potential to support adaptive and MRI-guided workflows; however, the small evidence base, methodological heterogeneity, and absence of independent external validation preclude conclusions regarding their comparative performance or clinical effectiveness (**Table 5**).

**Table 5:** Literature summary of AI-based image registration in HDR-IRT for cervical cancer.

Study	Design/Imaging	AI method	Target	Key findings
Ecker et al. (2022) (75)	Retrospective; MRI	3D UNETR and 2D U-Net	Applicator	3D UNETR outperformed 2D U-Net (DSC 0.70 vs 0.58). Registration achieved MDE 0.7 mm using ground-truth applicators, reducing workflow time from ~20 min to <30 s.
Salehi et al. (2023) (76)	Retrospective; CT	DIRNet CNN	CTV + OARs	DIRNet 4 achieved accurate deformable image registration (Dice 0.89–0.96, ASSD 1.06–1.61 mm) and outperformed conventional intensity-based registration across all evaluated organs.
Shrimanti et al. (2022) (77)	Retrospective; MRI	Inception V4-based CNN with autoencoder + modified U-Net	Uterus	Successfully predicted applicator-induced uterine deformation from pre-IRT MRI (DC 88.1%, HD 5.8 mm), enabling estimation of large uterine displacements (mean 25 mm).

2D, Two dimensional; 3D, Three dimensional; ASSD, average symmetric surface distance; CNN, convolutional neural network; CT, computed tomography; CTV, clinical target volume; DC, Dice coefficient; DSC, Dice similarity coefficient; HD, Hausdorff distance; HDR, high dose rate; IRT, interventional radiotherapy; MDE, mean distance error; mm, millimeter; MRI, magnetic resonance imaging; OARs, organs at risk; Retro, retrospective; s, second(s).

### 3.3 Risk of bias in studies and reporting bias

All studies included within each application category were assessable across the applicable methodological domains. The task-adapted QUADAS-2 assessment included 25 segmentation studies, seven reconstruction studies, and three image-registration studies. The PROBAST+AI 2025 assessment included 14 planning studies and four applicator-selection studies. Overall, the methodological assessment identified substantial risk-of-bias concerns across all five application domains, with the available evidence predominantly characterized by high risk of bias. The main recurrent limitations were retrospective single-center study designs, limited sample sizes, lack of independent external validation, non-independent reference standards, and, for prediction-oriented studies, potential overfitting or data leakage.

#### 3.3.1 Segmentation

The methodological assessment of the 25 segmentation studies identified an overall high risk of bias across the available evidence. Patient selection raised concerns in most studies (80.0%, n=20/25), mainly because of retrospective single-center designs and limited or selected datasets. The index-test domain represented the major source of bias, with 68.0% of studies (n=17/25) judged at high risk, primarily because of the lack of independent external validation and reliance on institution-specific datasets. The reference-standard domain also raised concerns in 72.0% of studies (n=18/25), particularly when ground-truth segmentations were generated by a single expert rather than through independent or consensus-based annotation. The flow-and-timing domain was judged at low risk of bias in all studies (100%, n=25/25). Overall, these methodological limitations indicate that the apparently high segmentation performance reported by individual studies should be interpreted cautiously, particularly about reproducibility and generalizability.

#### 3.3.2 Planning

According to the PROBAST+AI 2025 assessment, the overall risk of bias of planning studies was predominantly high. The participants and data sources domain raised concerns in most studies, with 78.6% (11/14) judged at intermediate risk of bias, mainly because of retrospective single-center datasets and limited sample sizes. The predictors domain was judged at low risk of bias in all studies (100%, 14/14), as predictors were typically objectively defined and consistently assessed. The outcome domain was judged at low risk of bias in 85.7% of studies (12/14), with outcomes generally based on predefined dosimetric or clinically established planning objectives. However, the analysis domain represented the major source of bias, particularly because of limited sample sizes, inadequate or insufficiently independent model evaluation, potential overfitting or data leakage, and the frequent absence of external validation. Consequently, favorable judgments in the predictors and outcome domains did not translate into a low overall risk of bias, and the reported performance estimates should be interpreted cautiously.

#### 3.3.3 Reconstruction

The methodological assessment of the reconstruction studies identified an overall high risk of bias across the available evidence. Patient selection raised concerns, mainly because of retrospective single-center designs and limited cohort sizes. The index-test domain represented the major source of bias, with most studies showing high or intermediate risk and no study including independent external validation. The reference standard was generally more robust, as ground truth was commonly derived from clinically approved treatment plans and expert annotations, while the flow-and-timing domain was consistently judged at low risk of bias. Despite favorable geometric and dosimetric performance reported by individual studies, the predominance of single-center datasets, limited sample sizes, and absence of independent external validation substantially restrict confidence in the reproducibility and generalizability of these findings.

#### 3.3.4 Applicator selection

According to PROBAST+AI 2025, the overall risk of bias of the applicator-selection studies was predominantly high. Concerns in the participants and data sources domain were mainly related to single-center designs and limited sample sizes. The predictors domain was generally judged at low risk of bias because predictors were objectively defined and reproducibly assessed, while the outcome domain was judged at low risk of bias in most studies. However, the analysis domain represented the principal source of bias, particularly because of limited sample sizes, insufficient independent evaluation, potential model overfitting, and the lack of external or multicenter validation. Therefore, despite promising reported discrimination and dosimetric performance, confidence in the robustness and generalizability of these models remains limited.

#### 3.3.5 Image registration

The methodological assessment of the three image-registration studies identified an overall high risk of bias across the available evidence. Patient selection raised concerns in all studies (100%, n=3/3), mainly because of retrospective single-center designs and limited sample sizes. The index-test domain represented the major methodological limitation and was judged at high risk of bias in all studies (100%, n=3/3), primarily because none performed independent external validation. The reference-standard domain was judged at low risk of bias in one study (33.3%, n=1/3) and raised concerns in two studies (66.7%, n=2/3), which relied on manual clinician delineations potentially affected by observer variability. The flow-and-timing domain was judged at low risk of bias in all studies. Overall, the high risk of bias, limited datasets, and absence of independent validation substantially restrict confidence in the reported registration performance and its generalizability across institutions and imaging systems.

### 3.4 Results of syntheses

The qualitative synthesis of 52 studies indicates that individual studies of AI-based image segmentation, treatment planning, and applicator reconstruction reported promising technical performance. Segmentation performance was variable across studies, with the highest-performing models reporting Dice Similarity Coefficients above 0.90 for selected organs at risk and above 0.85 for selected target volumes. Individual automated planning studies reported plan quality comparable to their respective conventional approaches, with reductions in organ-at-risk doses (0.6–4.3 Gy) and shorter optimization times reported in selected studies. Across application domains, the evidence consistently supported technical feasibility rather than demonstrated clinical benefit. The overall evidence base was characterized by substantial methodological heterogeneity, predominantly retrospective single-center designs, limited independent external validation, and reliance on technical or surrogate endpoints, preventing conclusions regarding clinical effectiveness or generalizability.

### 3.5 Certainty of evidence

The certainty of evidence ranged from Low to Very Low across the five application domains. Risk-of-bias concerns were primarily related to retrospective single-center designs, non-independent reference standards, potential data leakage or overfitting, and the limited use of independent external evaluation. Inconsistency arose from substantial variability in imaging modalities, anatomical structures, AI architectures, reference standards, and reported performance metrics. Indirectness was mainly attributable to reliance on geometric or dosimetric surrogate endpoints rather than patient-relevant clinical outcomes and to the use of institution-specific datasets. Imprecision reflected small study cohorts, limited independent test samples, and the frequent absence of confidence intervals or other measures of uncertainty. Publication bias was considered possible because most of the available evidence consisted of small retrospective studies reporting favorable AI performance, while negative or inconclusive studies may be underrepresented in the published literature. Publication bias was assessed qualitatively because the small number of studies within some application domains and the substantial heterogeneity of the reported outcomes and performance metrics precluded a reliable statistical assessment.

The final certainty rating for each application domain and the domain-specific reasons for downgrading are reported in **Supplementary Tables 1**-**5**. Correction of the risk-of-bias denominators did not change the final certainty ratings.

## 4 Discussion

This systematic review provides a comprehensive overview of artificial intelligence applications in image-guided cervical interventional radiotherapy, encompassing 52 studies across five major domains: segmentation (26–50), treatment planning (51–64), reconstruction (48, 65–70), applicator selection (71–74), and image registration (75–77). One study contributed to both segmentation and reconstruction analyses; therefore, the sum of studies across domains exceeds the total number of included studies (48). Overall, the available studies suggest that AI may improve selected aspects of workflow efficiency and standardization and may support treatment-related decision-making. However, the predominantly retrospective, single-center evidence base does not yet establish improvements in clinical outcomes or routine clinical effectiveness. The certainty of evidence was predominantly low to very low.

Studies investigated automated delineation of HR-CTV, CTV, GTV, OARs, and catheters using a wide range of architectures, including CNN, U-Net derivatives, transformer-based models, attention mechanisms, multimodal frameworks, and large language model-assisted approaches (26–50). Across studies, OAR segmentation frequently showed high reported performance, with Dice coefficients frequently exceeding 0.90 for bladder delineation and demonstrating high reported agreement with expert contours in individual studies (26, 30, 32–34, 40, 43, 49, 50). Target volume segmentation was more challenging because of tumor heterogeneity, anatomical distortion, and variations in MRI interpretation, although recent models such as SPSeg-Dual, MDA-TransUNet, Prompt-ResUNet, ARGNet, and AM-UNet reported promising technical performance (26, 35, 41, 43, 47).

These findings are consistent with broader evidence demonstrating the growing maturity of deep-learning-based auto-segmentation in radiation oncology (15, 16). Importantly, several studies reported substantial reductions in contouring time while maintaining dosimetric equivalence with manual contours, highlighting the potential clinical utility of AI-assisted contouring in image-guided interventional radiotherapy (26, 30, 36, 43, 49). Nevertheless, the task-adapted QUADAS-2 revealed important methodological concerns. Most studies were retrospective and single-center, while only a minority incorporated independent external validation cohorts (33–36). Consequently, the index test domain demonstrated a predominantly high risk of bias, reflecting uncertainty regarding generalizability across institutions, scanners, and contouring practices. These limitations contributed to the low-to-very-low certainty of evidence assigned to segmentation applications.

Treatment planning represented the second largest research area, comprising 14 studies focused on automated optimization, dose prediction, plan quality assurance, and adaptive planning (51–64). Collectively, these studies suggest that AI-based approaches can generate clinically acceptable plans with dosimetric characteristics comparable to expert-generated plans, while potentially reducing planning time. Several studies also reported improvements in OAR sparing without compromising target coverage (21, 53, 54, 56–60, 62, 63).

Among the approaches evaluated were the BRIGHT framework, which improved compliance with EMBRACE-II constraints (51), fully automated adaptive planning systems capable of producing physician-preferred plans (60, 62), and dose-prediction models achieving low prediction errors for both targets and OARs (57, 59, 64). These findings are consistent with broader developments in external beam radiotherapy, where AI-driven planning has been investigated as a potential approach to reduce planner variability and improve consistency.

Despite these encouraging findings, methodological limitations remain substantial. Most studies were retrospective and relied on institution-specific datasets, while only a limited number incorporated rigorous blinded validation procedures (51–64). As a result, the overall PROBAST+AI risk-of-bias judgment was predominantly high, despite more favorable assessments in the predictors and outcome domains. Consequently, the certainty of evidence was graded as very low, indicating that additional validation studies are required before routine clinical implementation.

Automated reconstruction studies focused primarily on applicator digitization, catheter localization, and interstitial needle reconstruction using CT- and MRI-based imaging (48, 65–70). Individual reconstruction studies reported favorable geometric performance, with submillimeter localization accuracy and minimal dosimetric deviations compared with expert manual reconstruction. Deep-learning-based methods, including attention-gated CNN, DSD-UNET architectures, and nnU-Net frameworks, reported Dice coefficients exceeding 0.88 in several studies (48, 65–70).

One study reported the substantial reduction in reconstruction time reported in MRI-guided workflows, where AI-assisted approaches reduced reconstruction times from approximately 46 minutes to 11 minutes without compromising accuracy (68). This observation is clinically relevant because applicator reconstruction remains one of the most technically demanding components of MRI-guided interventional radiotherapy.

However, the task-adapted QUADAS-2 assessment identified a recurrent methodological limitation: the complete absence of external validation. Although reconstruction tasks benefit from relatively objective reference standards derived from clinically approved treatment plans, most studies were conducted in single institutions and evaluated on limited datasets (48, 65–70). Consequently, despite the promising technical performance reported in individual studies, evidence certainty remained low.

Applicator selection represents an emerging potential application of AI in cervical interventional radiotherapy. The four included studies investigated machine-learning and deep-learning approaches to support personalized selection of applicator type, interstitial needle use, and template configuration (71–74). Deep-learning approaches reported promising classification performance for selecting between Syed and tandem-and-ovoid applicators, while machine-learning models successfully predicted the need for hybrid intracavitary-interstitial approaches and optimized needle placement strategies (71, 72). This domain included the first prospective clinical validation study identified in the review, conducted by Stenhouse et al. (74). This study reported lower OAR doses with AI-assisted decision support while maintaining target coverage, representing an important step toward clinical translation. Similarly, AI-guided 3D-printed template approaches improved HR-CTV coverage and reduced doses to critical organs compared with conventional free-hand insertion techniques (73).

Nevertheless, despite promising performance, evidence remains limited by the small number of studies and the absence of independent multicenter validation. The PROBAST+AI 2025 assessment identified methodological concerns in the participants and data sources and analysis domains, particularly because of the single-center study designs, limited sample sizes, and insufficient independent external validation, thereby restricting confidence in the generalizability of these decision-support systems.

Image registration was the least investigated domain, with only three studies addressing automated rigid registration, deformable image registration, and prediction of applicator-induced uterine deformation (75–77). Despite the limited evidence base, individual studies reported promising technical results. Deep-learning-based deformable registration reported higher performance than the conventional comparator evaluated within that study (75, 76). Furthermore, AI-based prediction of uterine deformation demonstrated the feasibility of estimating applicator-induced anatomical changes before interventional radiotherapy implantation (77).

These developments are particularly relevant in adaptive interventional radiotherapy, where anatomical variations and organ motion may influence cumulative dose delivery and treatment accuracy (12). Nevertheless, image registration studies consistently demonstrated high risk of bias within the index test domain due to the absence of external validation and the limited size of available datasets. Consequently, this remains one of the most exploratory fields within cervical IG-IRT, with very-low-certainty evidence despite promising initial results.

Taken together, the available evidence reveals a consistent pattern across the cervical IG-IRT workflow. AI applications have predominantly focused on automating technically demanding and operator-dependent tasks, with segmentation and treatment planning representing the most extensively investigated domains, whereas reconstruction, applicator selection, and image registration remain supported by smaller evidence bases. Across these applications, the most consistent findings concern technical feasibility, geometric or dosimetric agreement with conventional approaches, and potential reductions in workflow time or inter-observer variability. However, these findings have largely been generated in retrospective, single-center studies using heterogeneous datasets, imaging protocols, reference standards, and performance metrics. Consequently, although technical performance is promising across several application domains, the overall body of evidence remains insufficient to establish generalizability, clinical effectiveness, or superiority over established clinical workflows.

A consistent finding across all domains was the discrepancy between technical performance and clinical validation. Most studies reported geometric metrics such as Dice similarity coefficients, Hausdorff distances, localization errors, or classification accuracies, whereas relatively few investigated clinically meaningful endpoints including toxicity reduction, local control, treatment adaptation, physician acceptability, or patient outcomes. Although geometric accuracy is a prerequisite for clinical implementation, it does not necessarily translate into improved patient outcomes. Accordingly, high performance on geometric, dosimetric, or classification metrics should be interpreted as evidence of technical feasibility rather than evidence of clinical benefit. Demonstration of clinical benefit will require prospective evaluation using clinically meaningful endpoints, including workflow efficiency, treatment quality, toxicity, and oncologic outcomes.

The methodological quality assessments further highlighted common weaknesses. Across segmentation, reconstruction, and registration studies, task-adapted QUADAS-2 assessment consistently identified a high risk of bias in the index test domain due to the absence of independent external validation. Similarly, the PROBAST+AI 2025 assessments of planning and applicator-selection studies identified recurrent concerns in the analysis domain, particularly regarding external validation and model generalizability. These findings explain the predominance of low-to-very-low GRADE ratings observed throughout the review. Explainability represents an additional challenge for clinical translation. Many of the investigated approaches, particularly deep-learning models, generate predictions without providing clinicians with transparent information on the features or decision processes underlying their outputs. This may limit clinician confidence and complicate the identification of model failures, particularly in high-stakes tasks such as target delineation, treatment planning, and applicator selection. Future studies should therefore incorporate interpretable outputs and clinically meaningful quality-assurance strategies that allow clinicians to understand, verify, and, when necessary, override AI-generated recommendations. Beyond model performance and explainability, successful clinical implementation will require integration into existing treatment-planning systems and clinical workflows, standardized quality-assurance procedures, and clear definition of the role of clinician oversight. Performance should also be evaluated across different institutions, imaging devices, acquisition protocols, and patient populations to determine whether models remain reliable outside the environments in which they were developed. These implementation requirements are particularly relevant in IG-IRT, where AI-generated outputs may directly influence target delineation, treatment planning, applicator reconstruction, or procedural decision-making (21, 22).

The main strength of this review is its comprehensive assessment of AI applications throughout the entire cervical IG-IRT workflow. To our knowledge, this is the first systematic review simultaneously evaluating segmentation, planning, reconstruction, applicator selection, and image registration within a single framework. Furthermore, the study incorporates task-specific risk-of-bias tools (QUADAS-2, AI-specific considerations proposed within the QUADAS-AI initiative, and PROBAST+AI 2025) together with a GRADE-based certainty assessment, providing a robust evaluation of methodological quality and clinical maturity.

Several limitations should also be acknowledged. First, substantial heterogeneity existed among studies regarding imaging modalities, AI architectures, datasets, and performance metrics, precluding quantitative meta-analysis. Second, most included studies were retrospective and single-center investigations. Third, publication bias cannot be excluded because studies reporting favorable AI performance are more likely to be published. Finally, the rapidly evolving nature of AI research means that emerging algorithms may quickly modify the current evidence landscape.

## 5 Conclusions

The available evidence suggests that artificial intelligence may support the automation of selected labor-intensive tasks, reduce inter-observer variability, and assist treatment-related decision-making in image-guided cervical interventional radiotherapy. Segmentation was the most frequently investigated application and therefore had the largest evidence base, but substantial heterogeneity and limited external validation prevent conclusions regarding the relative superiority or clinical maturity of individual approaches. Planning and reconstruction studies also reported promising technical and workflow outcomes, whereas applicator selection and image registration remain less extensively investigated. At present, no AI approach identified in this review can be considered ready for routine clinical implementation. Across all domains, robust prospective multicenter evaluation, independent external validation, standardized evaluation methods, and assessment of clinically meaningful endpoints are required before widespread clinical adoption. Future research should prioritize standardized evaluation frameworks, multicenter collaborations, and clinically meaningful endpoints to establish the real-world impact of AI in cervical interventional radiotherapy.

## Supplementary material

Supplementary Table 1 Summary of findings segmentation.

Supplementary Table 2 Summary of findings planning.

Supplementary Table 3 Summary of findings reconstruction.

Supplementary Table 4 Summary of findings applicator selection.

Supplementary Table 5 Summary of findings image registration.

Supplementary Table 6 Domains, task-specific signalling questions, and decision criteria used for the methodological assessment.
