# Supplementary material for: Artificial intelligence in image-guided cervical interventional radiotherapy: a systematic review
Source: Front Oncol. 2026 Oct 15;16:1910070. doi: 10.3389/fonc.2026.1910070 (PMC13716582; doi:10.3389/fonc.2026.1910070)
Supplement: Supplementary file 1 [file Table1.docx]

| Study design | Risk of bias | Inconsistency | Indirectness | Imprecision | N. of patients | Effect | | Certainty |
| --- | --- | --- | --- | --- | --- | --- | --- | --- |
| **Bladder Overlap** | | | | | | | | |
| Observational | Not serious | Not serious | Not serious | Not serious | 1500 scans | | Median DSC: 0.90 – 0.93 \| Median HD95: < 3.2 mm | Low |
| **Rectum Overlap** | | | | | | | | |
| Observational | Not serious | Not serious | Not serious | Serious^a^ | 1100 scans | | Median DSC: 0.83 – 0.88 \| Median HD95: < 4.1 mm | Very low |
| **Sigmoid Overlap** | | | | | | | | |
| Observational | Not serious | Serious^b^ | Not serious | Serious^c^ | 850 scans | | Median DSC: 0.60 – 0.78 \| High boundary variance | Very low |
| **Target Volume** | | | | | | | | |
| Observational | Serious^d^ | Serious^e^ | Not serious | Not serious | 1900 scans | | Median DSC: 0.74 – 0.92 \| Highly dependent on MRI/CT contrast | Very low |
| **Hardware & Needles** | | | | | | | | |
| Observational | Serious^f^ | Not serious | Not serious | Not serious | 2500 channel | | Tip Error: 0.5 – 1.1 mm \| Median DSC: 0.88 – 0.93 | Very low |

***Abbreviations****: DSC: Dice Similarity Coefficient; HD95: 95th percentile Hausdorff Distance; MRI: Magnetic Resonance Imaging; CT: Computed Tomography.*

**Explanations:**

a: small verification population or restricted hold-out testing sample

b: high anatomical variation of the sigmoid colon across fractions

c: small verification population or restricted hold-out testing sample

d: significant risk of inter-observer contouring variability across baseline registries

e: high performance variance observed in smaller or regressed target volumes

f: pervasive absence of independent external validation on multi-vendor scanner data
