# Supplementary material for: Artificial intelligence in image-guided cervical interventional radiotherapy: a systematic review
Source: Front Oncol. 2026 Oct 15;16:1910070. doi: 10.3389/fonc.2026.1910070 (PMC13716582; doi:10.3389/fonc.2026.1910070)
Supplement: Supplementary file 2 [file Table2.docx]

| Study design | Risk of bias | Inconsistency | Indirectness | Imprecision | N. of patients | Effect | | Certainty |
| --- | --- | --- | --- | --- | --- | --- | --- | --- |
| **Dosimetric Target Quality** | | | | | | | | |
| Observational | Not serious | Not serious | Not serious | Not serious | 2800 fractions | | Non-inferior target coverage \| Mean ΔD _90%_ stable (± 0.5%) | Low |
| **Dosimetric OAR Sparing** | | | | | | | | |
| Observational | Not serious | Not serious | Not serious | Not serious | 3100 fractions | | Mean Δ2cc reduction up to 10% (0.6–4.3 Gy) | Low |
| **Clinical Acceptability** | | | | | | | | |
| Observational | Not serious | Not serious | Not serious | Serious^a^ | ~150 plans | | Independent clinician preference/indistinguishability rates range between 50.8% and 75.0%–76.3% | Very low |
| **Optimization Efficiency** | | | | | | | | |
| Observational | Serious^b^ | Not serious | Not serious | Not serious | ~2200 runs evaluated | | manual ~60 minutes versus range 20 seconds-3.5 minutes under AI deployment. | Very low |
| **Plan Approval Probability** | | | | | | | | |
| Observational | Serious^C^ | Not serious | Serious^d^ | Not serious | 218 fractions | | AUC range 0.74-0.95 | Very low |

***Abbreviations****: AUC: Area Under the Curve*

**Explanations:**

a: restricted patient numbers in selected blinded clinical testing sets: 10 to 28 cases

b: severe fraction-level data partitioning inducing inter-fraction data leakage in several architectures

c: high training dataset inconsistency due to historical unoptimized clinical plans in archives

d: utilizing artificially perturbed plans to synthesize negative data samples
