# Supplementary material for: Artificial intelligence in image-guided cervical interventional radiotherapy: a systematic review
Source: Front Oncol. 2026 Oct 15;16:1910070. doi: 10.3389/fonc.2026.1910070 (PMC13716582; doi:10.3389/fonc.2026.1910070)
Supplement: Supplementary file 3 [file Table3.docx]

| Study design | Risk of bias | Inconsistency | Indirectness | Imprecision | N. of patients | Effect | | Certainty |
| --- | --- | --- | --- | --- | --- | --- | --- | --- |
| **Centerline Extraction** | | | | | | | | |
| Observational | Not serious | Not serious | Not serious | Not serious | ~120 rigid applicators | | Mean 3D path error: 0.30 – 0.97 mm. | Low |
| **Tip Localization Accuracy** | | | | | | | | |
| Observational | Serious^a^ | Serious^b^ | Not serious | Not serious | ~380 needles | | Longitudinal tip error: 0.63 – 1.10 mm. | Very low |
| **MR-Only Semiautomatic Path** | | | | | | | | |
| Observational | Not serious | Not serious | Not serious | Serious^c^ | 88 catheters | | Mean path error: 0.97 ± 0.66 mm \| Reconstruction time: 11 mins | Very low |

***Abbreviations****: AUC: Area Under the Curve*

**Explanations:**

a: thick slice acquisitions 3–5 mm causing voxel partial volume effects

b: increased error variance at the distal edge due to heavy metal streaking artifacts

c: very small validation population
