# Supplementary material for: Artificial intelligence in image-guided cervical interventional radiotherapy: a systematic review
Source: Front Oncol. 2026 Oct 15;16:1910070. doi: 10.3389/fonc.2026.1910070 (PMC13716582; doi:10.3389/fonc.2026.1910070)
Supplement: Supplementary file 4 [file Table4.docx]

| Study design | Risk of bias | Inconsistency | Indirectness | Imprecision | N. of patients | Effect | | Certainty |
| --- | --- | --- | --- | --- | --- | --- | --- | --- |
| **Technical Applicator Classification** | | | | | | | | |
| Observational | Not serious | Not serious | Not serious | Not serious | 422 insertions | | accuracy 96.0%, specificity 97.4%, sensitivity of 90.9%. | Low |
| **IRT Technique Selection** | | | | | | | | |
| Observational | Not serious | Not serious | Not serious | Serious^a^ | ~169 fractions | | Selection between ICIRT and hybrid IC/ISIRT achieved 84.9% accuracy and 70.0% prospective accuracy reducing OAR dose by 2.0 Gy mean. | Very low |
| **Template Path Configuration** | | | | | | | | |
| Observational | Not serious | Not serious | Not serious | Serious^a^ | 17 patients | | 3D-printed template automated path generation completed in 6 mins, with 82.5% needle selection accuracy and zero OAR intersections. | Very low |

***Abbreviations****: DSC: ICBT: Intracavitary Brachytherapy; IC/ISBT: Hybrid Intracavitary/Interstitial Brachytherapy; OAR: Organ At Risk.*

**Explanations:**

a: very small testing sample size or narrow clinical validation co-cohort
