# Supplementary material for: Artificial intelligence in image-guided cervical interventional radiotherapy: a systematic review
Source: Front Oncol. 2026 Oct 15;16:1910070. doi: 10.3389/fonc.2026.1910070 (PMC13716582; doi:10.3389/fonc.2026.1910070)
Supplement: Supplementary file 5 [file Table5.docx]

| Study design | Risk of bias | Inconsistency | Indirectness | Imprecision | N. of patients | Effect | | Certainty |
| --- | --- | --- | --- | --- | --- | --- | --- | --- |
| **Rigid Inter-fraction MDE** | | | | | | | | |
| Observational | Not serious | Not serious | Not serious | Serious^a^ | 20 fractions | | MDE: 2.7 ± 1.4 mm \| Latency < 30s | Very low |
| **Deformable Organ Mapping** | | | | | | | | |
| Observational | Not serious | Not serious | Serious^B^ | Serious^a^ | ~380 needles | | Needle tip longitudinal error: 0.63 – 1.10 mm | Very low |
| **Predictive Uterine DIR** | | | | | | | | |
| Observational | Not serious | Not serious | Not serious | Serious^a^ | 92 patients | | DSC: 88.1 ± 3.8 \| HD: 5.8 ± 3.6 mm | Very low |

***Abbreviations****: DSC: Dice Similarity Coefficient; MDE: Mean Distance Error*

**Explanations:**

a: very small testing sample size or narrow clinical validation co-cohort

b: utilizing artificial Gaussian stochastic fields to simulate deformations
