# Supplementary material for: Artificial intelligence in image-guided cervical interventional radiotherapy: a systematic review
Source: Front Oncol. 2026 Oct 15;16:1910070. doi: 10.3389/fonc.2026.1910070 (PMC13716582; doi:10.3389/fonc.2026.1910070)
Supplement: Supplementary file 6 [file Table6.docx]

| **Framework** | **Domain** | **Task-specific signalling questions** | **Decision criteria** |
| --- | --- | --- | --- |
| **Adapted QUADAS-2** | **Patient selection** | **Was the source, size, and quality of input data accurately characterized alongside clear patient eligibility criteria? Was the dataset derived from open-source repositories? Was the rationale and breakdown of training, validation, and test sets reported? Was image preprocessing performed/reported? Was information on the scanner/model used for image acquisition provided?** | **Low/high/unclear** |
| **Adapted QUADAS-2** | **Index test** | **Was external validation performed?** | **Low/high/unclear** |
| **Adapted QUADAS-2** | **Reference standard** | **Was the reference standard likely to correctly classify the target condition?** | **Low/high/unclear** |
| **Adapted QUADAS-2** | **Flow and timing** | **Was the time interval between the index test and reference standard appropriate?** | **Low/high/unclear** |
| **PROBAST+AI-** **2025** | **Participants/data sources** | **Were appropriate data sources used? Was an appropriate study design used? Did the inclusions and exclusions of study participants result in a representative dataset?** | **Low/high/unclear** |
| **PROBAST+AI-** **2025** | **Predictors** | **Were predictors defined and assessed in a similar way for all participants? Was any preprocessing of predictors similar for all participants? Were predictor assessments made without knowledge of outcome data? Were the predictors included in the model available at the time the model was intended to be used?** | **Low/high/unclear** |
| **PROBAST+AI-** **2025** | **Outcome** | **Were outcomes defined and assessed appropriately? Were outcomes defined and assessed in a similar way for all participants? Were outcome assessments made without use or knowledge of predictor data? Was the time interval between predictor assessment and outcome assessment appropriate?** | **Low/high/unclear** |
| **PROBAST+AI-2025** | **Analysis** | **Model development**  **Was there evidence that the sample size was reasonable? Were continuous and categorical predictors handled appropriately? Were participants with missing or censored data handled appropriately in the analysis? If methods to address class imbalance were used, was the model or the model predictions recalibrated? Were methods used to address potential model overfitting?**  **Model evaluation**  **Was model evaluation based on only apparent performance avoided? Was there evidence that the sample size was reasonable? Were participants with missing or censored data handled appropriately in the analysis? If methods to address class imbalance were used, was the evaluation done in a dataset without correction for imbalance? If data splitting was done to create training and test datasets, was there evidence that data leakage was avoided? If resampling methods were used to evaluate model performance, were all model development steps replicated in the resampling process? Was the predictive performance of the model evaluated appropriately—for example, calibration, discrimination, and net benefit?** | **Low/high/unclear** |
