# Supplementary material for: Artificial intelligence in image-guided cervical interventional radiotherapy: a systematic review
Source: Front Oncol. 2026 Oct 15;16:1910070. doi: 10.3389/fonc.2026.1910070 (PMC13716582; doi:10.3389/fonc.2026.1910070)
Supplement: Supplementary file 7 [file Table7.docx]

| **Database** | **Complete search strategy** | **Limits** |
| --- | --- | --- |
| PubMed/MEDLINE | ( "cervical cancer" OR "cervix cancer" OR "cervical neoplasm" OR "cervical neoplasms" OR “cervix neoplasm” OR “cervix neoplasms”) AND (“ brachytherapy” OR "image guided brachytherapy" OR "image-guided brachytherapy" OR "interventional radiotherapy") AND ( “MRI” OR "magnetic resonance imaging" OR “CT” OR "computed tomography" OR “imaging”) AND ( "machine learning" OR "artificial intelligence" OR "deep learning" OR "neural network" OR "neural networks") | 1 January 2010–31 May 2026; no language filter |
| Scopus | TITLE-ABS-KEY(("cervical cancer" OR "cervix cancer" OR "cervical neoplasm" OR "cervical neoplasms") AND (brachytherapy OR "image guided brachytherapy" OR "interventional radiotherapy") AND (MRI OR "magnetic resonance imaging" OR CT OR "computed tomography" OR imaging) AND ("machine learning" OR "artificial intelligence" OR "deep learning" OR "neural network" OR "neural networks")) | 1 January 2010–31 May 2026; no language filter |
| Web of Science Core Collection | ("cervical cancer" OR "cervix cancer" OR "cervical neoplasm" OR "cervical neoplasms") AND (brachytherapy OR "image guided brachytherapy" OR "image-guided brachytherapy" OR "interventional radiotherapy") AND (MRI OR "magnetic resonance imaging" OR CT OR "computed tomography" OR imaging) AND ("machine learning" OR "artificial intelligence" OR "deep learning" OR "neural network" OR "neural networks") | 1 January 2010–31 May 2026; no language filter |
