# Supplementary material for: Artificial intelligence in image-guided cervical interventional radiotherapy: a systematic review
Source: Front Oncol. 2026 Oct 15;16:1910070. doi: 10.3389/fonc.2026.1910070 (PMC13716582; doi:10.3389/fonc.2026.1910070)
Supplement: Supplementary file 8 [file Table8.docx]

| **No.** | **Study** | **Reason for exclusion** |
| --- | --- | --- |
| 1 | **Deep learning and dual-radiomics model incorporating brachytherapy applicator type to predict radiation-induced acute rectal injury in cervical cancer patients** | The study predicts radiation-induced acute rectal injury after radiotherapy using CT radiomics, dosiomics and DL. Although applicator type is incorporated as a variable, the AI model is not applied to interventional radiotherapy imaging, target/OAR segmentation, registration, treatment planning, or applicator/needle guidance. |
| 2 | **Machine Learning-Based Prognostic Modelling Using MRI Radiomic Data in Cervical Cancer Treated with Definitive Chemoradiotherapy and Brachytherapy** | The study applies machine learning to pre-treatment MRI-derived radiomic features for survival and outcome prediction, including distant metastasis-free survival. Although patients received interventional radiotherapy as part of their treatment, AI was not applied to the interventional radiotherapy procedure, imaging, treatment planning, or delivery. Furthermore, radiomics-based applications were outside the scope of the present review and were not included in the analysis. |
| 3 | **Delta-radiomics analysis based on magnetic resonance imaging to identify radiation proctitis in patients with cervical cancer after radiotherapy** | The study applies MRI-based delta-radiomics and machine learning to predict the severity of radiation proctitis after radiotherapy. The AI application is not specifically related to cervical interventional radiotherapy imaging, segmentation, registration, treatment planning, or delivery. Furthermore, radiomics-based applications were outside the scope of the present review and were not included in the analysis. |
| 4 | **Development, validation, and visualization of a novel nomogram for predicting clinical outcomes of radiotherapy combined with chemotherapy in locally advanced cervical cancer** | AI/radiomics is used to predict local recurrence or distant metastasis after chemoradiotherapy from DWI and clinical variables. There is no interventional radiotherapy-specific AI application described. |
| 5 | **Longitudinal dynamic MRI radiomic models for early prediction of prognosis in locally advanced cervical cancer treated with concurrent chemoradiotherapy** | Although MRI is acquired before interventional radiotherapy (MRImid), the ML model uses longitudinal radiomics to predict 2-year PFS and OS. The AI application is prognostic rather than an application to interventional radiotherapy segmentation, registration, planning or delivery. |
| 6 | **Integrating Novel and Classical Prognostic Factors in Locally Advanced Cervical Cancer: A Machine Learning-Based Predictive Model (ESTHER Study)** | Although all patients received chemoradiation and interventional radiotherapy, ML is applied to clinical, nutritional, inflammatory and body-composition variables for outcome prediction (LC, MFS, DFS, OS), not to imaging or the brachytherapy workflow. |
| 7 | **Machine learning-based radiomics for predicting outcomes in cervical cancer patients undergoing concurrent chemoradiotherapy** | The study applies MRI-based radiomics and machine learning to predict disease-free survival (DFS) and overall survival (OS) after concurrent chemoradiotherapy. The abstract does not describe an AI application specifically related to interventional radiotherapy imaging, segmentation, registration, treatment planning, or delivery. Furthermore, radiomics-based applications were outside the scope of the present review and were not included in the analysis. |
| 8 | **Clinical Workflow of Cone Beam Computer Tomography-Based Daily Online Adaptive Radiotherapy with Offline Magnetic Resonance Guidance: The Modular Adaptive Radiotherapy System (MARS)** | This concerns machine-learning-supported online adaptive external beam radiotherapy (Ethos), not interventional radiotherapy. Moreover, only 5/31 patients had cervical cancer, and results are not reported separately for them. |
| 9 | **Daily AI-Based Treatment Adaptation under Weekly Offline MR Guidance in Chemoradiotherapy for Cervical Cancer: The AIM-C1 Trial** | AI is used for CBCT-guided online adaptive EBRT. Interventional radiotherapy (28 Gy/4 fractions) is subsequently administered as standard treatment but is not the object of the AI intervention. |
| 10 | **Bladder Segmentation in MRI for High Dose Rate Brachytherapy Using Deep Network** | The deep-learning model was developed and evaluated exclusively for bladder segmentation and did not include the other organs at risk required by the predefined eligibility criteria; therefore, the study was excluded. |
| 11 | **The accuracy of artificial intelligence deformed nodal structures in cervical online cone-beam-based adaptive radiotherapy** | The study evaluates artificial intelligence-guided deformation for automatic nodal CTV generation and treatment adaptation during online cone-beam CT-based external beam adaptive radiotherapy in patients with cervical cancer. Although AI-based contouring and dosimetric performance were assessed, the application is exclusively related to external beam radiotherapy and not to interventional radiotherapy/brachytherapy imaging, segmentation, treatment planning, or delivery. |
| 12 | **Feasibility and Clinical Acceptability of Automation-Assisted 3D Conformal Radiotherapy Planning for Patients with Cervical Cancer in a Resource-Constrained Setting** | The study evaluates an automation-assisted system for contouring and treatment planning in 3D conformal external beam radiotherapy for cervical cancer. Although automated segmentation of the primary tumor, nodal CTVs, and organs at risk and automated treatment planning were assessed, the application is exclusively related to external beam radiotherapy and not to interventional radiotherapy/brachytherapy imaging, segmentation, treatment planning, or delivery. |
| 13 | **Implementing cone-beam computed tomography-guided online adaptive radiotherapy in cervical cancer** | The study evaluates AI-driven OAR contouring and automated treatment planning within a CBCT-guided online adaptive external beam radiotherapy workflow for locally advanced cervical cancer. Although artificial intelligence is used for automated contour delineation and adaptive treatment planning, the application is exclusively related to external beam radiotherapy and not to interventional radiotherapy/brachytherapy imaging, segmentation, treatment planning, or delivery. |
| 14 | **Automatic landmark correspondence detection in medical images with an application to deformable image registration** | The study develops a deep-learning method for automatic landmark detection to improve deformable image registration in three-dimensional medical images. Although the method was tested on CT scans from patients with cervical cancer, the study does not specifically address interventional radiotherapy/brachytherapy or demonstrate an application of the proposed image-registration approach to interventional radiotherapy imaging, treatment planning, or delivery. |
| 15 | **Convolutions, Transformers, and their Ensembles for the Segmentation of Organs at Risk in Radiation Treatment of Cervical Cancer** | The study compares different deep-learning architectures and ensemble approaches for automatic segmentation of multiple organs at risk (bladder, bowel, rectum, and sigmoid) on MRI scans of patients with cervical cancer. However, the abstract does not identify the imaging dataset or segmentation task as specifically related to interventional radiotherapy/brachytherapy, nor does it demonstrate an application to interventional radiotherapy treatment planning or delivery. |
| 16 | **A knowledge-based organ dose prediction tool for brachytherapy treatment planning of patients with cervical cancer** | The study investigates knowledge-based OAR dose estimation for CT-based intracavitary cervical interventional radiotherapy, using HR-CTV boundary-distance subvolumes and cohort-averaged differential DVHs to predict bladder, rectum, and sigmoid D2cc. However, the methodology does not involve artificial intelligence, machine learning, or deep-learning algorithms; therefore, the study was outside the scope of the present review. |

*Abbreviation:* 3D, three-dimensional; AI, artificial intelligence; CBCT, cone-beam computed tomography; CT, computed tomography; CTV, clinical target volume; D2cc, minimum dose received by the most exposed 2 cm³; DFS, disease-free survival; DL, deep learning; DWI, diffusion-weighted imaging; EBRT, external beam radiotherapy; HR-CTV, high-risk clinical target volume; LC, local control; MARS, Modular Adaptive Radiotherapy System; MFS, metastasis-free survival; ML, machine learning; MRI, magnetic resonance imaging; OAR, organ at risk; OS, overall survival; PFS, progression-free survival.
